# Editorial: Music, Brain, and Rehabilitation: Emerging Therapeutic Applications and Potential Neural Mechanisms

**DOI:** 10.3389/fnhum.2016.00103

**Published:** 2016-03-09

**Authors:** Teppo Särkämö, Eckart Altenmüller, Antoni Rodríguez-Fornells, Isabelle Peretz

**Affiliations:** ^1^Cognitive Brain Research Unit, Institute of Behavioural Sciences, University of HelsinkiHelsinki, Finland; ^2^Institute of Music Physiology and Musicians' Medicine, University of Music, Drama and Media HanoverHanover, Germany; ^3^Cognition and Brain Plasticity Unit, Bellvitge Research Biomedical InstituteBarcelona, Spain; ^4^Department of Basic Psychology, University of BarcelonaBarcelona, Spain; ^5^Institució Catalana de Recerca i Estudis AvançatsBarcelona, Spain; ^6^International Laboratory for Brain, Music, and Sound Research and Centre for Research on Brain, Language and MusicMontréal, QC, Canada; ^7^Department of Psychology, Université de MontréalMontréal, QC, Canada

**Keywords:** music, cognition, movement, brain, neurological disorders, rehabilitation, neuroimaging

Music is an important source of enjoyment, learning, and well-being in life as well as a rich, powerful, and versatile stimulus for the brain. With the advance of modern neuroimaging techniques during the past decades, we are now beginning to understand better what goes on in the healthy brain when we listen, play, think, and feel music and how the structure and function of the brain can change as a result of musical training and expertise. In the healthy brain, there is already mounting evidence that a large-scale bilateral network of temporal, frontal, parietal, cerebellar, and limbic/paralimbic brain areas associated with auditory perception, language, syntactic and semantic processing, attention and working memory, semantic and episodic memory, rhythmic and motor functions, and emotions and reward underlies the processing of music (Koelsch, 2011, 2014; Zatorre and Salimpoor, 2013; Janata, 2015) and to which extent this neural network could be shaped by musical training (Kraus and Chandrasekaran, 2010; Herholz and Zatorre, 2012; Brown et al., 2015). In the field of neurology, music has traditionally been studied in the context of musical deficits (e.g., amusia; Peretz et al., 2003), music-related symptoms (e.g., musicogenic epilepsy; Maguire, 2015), cases of exceptional or preserved musical functions (e.g., singing in aphasia; Johnson and Graziano, 2015), and neurological disorders of professional musicians (e.g., musician's dystonia; Altenmüller et al., 2015).

During the last decade, there has been increasing interest and progress in adopting music as a therapeutic tool in neurological rehabilitation, and many novel music-based methods have been developed to improve motor, cognitive, language, emotional, and social deficits in persons suffering from a debilitating neurological illness, ranging from childhood and adolescence [e.g., autism (Geretsegger et al., 2014), dyslexia (Flaugnacco et al., 2015)] to adulthood and old age [e.g., stroke (Särkämö et al., 2008; Bradt et al., 2010; Rodríguez-Fornells et al., 2012; Altenmüller and Schlaug, 2015), Parkinson's disease (Nombela et al., 2013; Bloem et al., 2015), and dementia (Vink et al., 2011; Baird and Samson, 2015)]. Traditionally, the fields of music neuroscience and music therapy have progressed independently, providing separate lines of evidence for how music is processed in the healthy brain and how it can be used therapeutically. We are now finally reaching a point where these fields are starting to merge and integrate, providing novel and important information about how music is processed in the damaged or abnormal brain, how structural and functional recovery of the brain can be enhanced by music-based rehabilitation methods, and what neural mechanisms underlie the therapeutic effects of music (for a related discussion, see Magee and Stewart). In the future, this information is pivotal for increasing our understanding of how and why music works in rehabilitation and for developing more effective music-based applications that are better targeted at specific brain processes and better tailored toward the individual rehabilitation needs of patients.

With these goals in mind, we launched the current Research Topic, jointly hosted by Frontiers in Human Neuroscience and Frontiers in Auditory Cognitive Neuroscience, which aimed to bring together research across multiple disciplines with a special focus on music, brain, and neurological rehabilitation. We invited researchers to present research addressing either how musical skills and attributes, such as music perception, experiencing music emotionally, or playing or singing, are affected by a developmental or acquired neurological disorder or what is the applicability, effectiveness, and mechanisms of music-based rehabilitation methods in neurological patients.

We were delighted that our call was met with enthusiasm and was answered by many research groups across the world, resulting in altogether 27 papers published in Frontiers in Human Neuroscience (21 papers) and Frontiers in Auditory Cognitive Neuroscience (six papers). Twenty-three papers were Original Research Articles, three were Reviews, and one was a General Commentary. There were altogether 132 authors from 14 countries (Australia, Canada, China, Cuba, Denmark, Finland, France, Germany, Italy, Netherlands, Poland, Spain, UK, and USA), providing an interesting cross-section to the global state-of-the-art on research currently done in the field of music, neuroscience, and neurorehabilitation. Broadly classified, the papers focused on six core topics: (i) music and hearing impairment; (ii) music, rhythm, and language; (iii) music, rhythm, and movement; (iv) music, learning, and memory; (v) responsiveness to music in severe neurological disorders; and (vi) novel sound-based technological advances. Next, we will provide a brief overview of these studies.

## Music and hearing impairment

Four papers presented novel research related to deafness and cochlear implants (CIs), auditory prostheses that restore hearing ability via electrical stimulation of the auditory nerve. Due to the spectrotemporally degraded nature of the sound transmission, CI users typically face many challenges in more complex listening tasks, such as when perceiving music. Petersen et al. report an EEG study where they compared adolescent, prelingually deaf CI users and normal-hearing controls for their mismatch negativity (MMN) responses to different auditory changes (pitch, timbre, intensity, and rhythm) in a musical melodic context. Compared to the healthy controls, the MMN responses were smaller in CI users. Especially the MMN to pitch changes was diminished in CI users, whereas they showed significant MMNs to timbre, rhythm, and intensity changes. Using the same musical multi-feature paradigm and EEG, Timm et al. compared adult CI users, who were postlingually deafened and late-implanted, with normal-hearing controls. The adult CI users showed abolished MMNs to complex rhythmic changes as well as smaller and later MMNs to pitch changes, whereas they elicited MMNs comparable to controls for timbre and intensity changes. Together, these findings indicate that although both pre- and postlingually deaf CI users have clear pitch discrimination difficulties, their brains are nevertheless able to extract more musically relevant information from sound than previously thought, making music-based interventions a viable tool for CI users.

The impact of musical training on auditory perceptual and cognitive performance in deaf children (with CIs or hearing aids) was studied by Rochette et al. Utilizing an innovative interactive game, they compared auditory discrimination, identification, scene analysis, and working memory as well as phonetic discrimination between deaf children who had previously received music lessons for 1.5–4 years and control deaf children who had not received music lessons. The musically trained children showed better performance in auditory scene analysis, auditory working memory, and phonetic discrimination tasks than the non-trained children, suggesting that musical training in deaf children contributes to the development of auditory attention and perception, which, in turn, can facilitate auditory-related cognitive and linguistic skills. The link between musical training and perception of degraded pitch was also studied by Fuller et al. They compared normal hearing musicians and non-musicians on tasks involving speech, vocal emotion, and melodic contour identification under normal and CI simulation listening conditions. Better performance in musicians was observed for vocal emotion and melodic contour identification in both conditions and for word identification only in the CI condition. Overall, this musician effect was stronger as the importance of pitch in the task increased, suggesting that musical training can be beneficial especially for challenging pitch perception, as in the case of the CI.

## Music, rhythm, and language

The close linkage between music, rhythm, and language, especially in the context of reading and speech production impairments, was explored in four papers. Flaugnacco et al. evaluated a group of dyslexic children with an extensive battery of neuropsychological tests, phonological tasks, and psychoacoustic and musical tasks. Results indicated a strong link between several temporal skills, such as meter perception and rhythm reproduction, and phonological and reading abilities, encouraging the use of music training, especially focused on rhythm, as rehabilitative tool in dyslexic children. Zumbansen et al. performed a cross-over study in three stroke patients with Broca's aphasia aimed at evaluating the relative contribution of rhythm and pitch on the effectiveness of *melodic intonation therapy* (MIT), a structured singing-based rehabilitation protocol for aphasia. They assessed connected speech, speech accuracy of trained and non-trained sentences, motor-speech agility, and mood before and after receiving melodic therapy (with pitch and rhythm), rhythmic therapy (with rhythm only), and normal spoken therapy. The results showed that whereas all treatments improved speech accuracy in trained sentences, the melodic therapy elicited the strongest generalization effect both to non-trained stimuli and to connected speech, underscoring the importance of both rhythm and pitch components in MIT.

The roles of the different components of MIT and its underlying mechanisms were also discussed by Merrett et al. In their comprehensive review of MIT literature, they identified four mechanisms potentially underlying the efficacy of MIT: neuroplastic reorganization of language function, activation of the mirror neuron system and multimodal integration, utilization of shared or specific features of music and language, and motivation and mood. These mechanisms are not mutually exclusive, but reflect the neurobiological, cognitive, and emotional effects of MIT, and together contribute to the efficacy of the therapy. Fujii and Wan reviewed studies about the role of rhythm in music and in speech perception and production and their rehabilitation. With an aim of explaining how and why musical rhythm can benefit speech and language rehabilitation, they propose a novel *SEP hypothesis* postulating that “sound envelope processing” and “synchronization and entrainment to pulse” may help stimulate different brain networks, including auditory afferent, subcortical-prefrontal, striato-thalamo-cortical, and cortical motor efferent circuits, which underlie human communication.

## Music, rhythm, and movement

Five papers discussed the links between music, rhythm, and movement in the healthy brain and in Parkinson's disease (PD) and stroke patients. Using fMRI in healthy subjects, Schaefer et al. studied how imagined or musical cueing changes the way the motor system is activated during simple movements. Moving to real music increased the activation in specific cerebellar areas whereas moving to imagined music activated especially pre-supplementary and basal ganglia motor areas, indicating that these two types of cueing have a different neural basis. Leow et al. explored the impact of different auditory cues, varying in their beat salience and musical nature, on the ability to synchronize one's movements to the auditory rhythm. In a behavioral experiment, they showed that high-groove music was superior to low-groove music in synchronizing gait and in eliciting longer and faster steps and that low-groove music was particularly detrimental to gait in weak beat-perceivers, indicating that both beat salience and beat perception skills are important mediators in movement cueing.

Auditory cueing can improve gait in PD patients (Nombela et al., 2013; Bloem et al., 2015). Benoit et al. extended this finding by determining whether *auditory-cued gait training* with music can facilitate both motor and perceptual timing in PD patients. Indeed, the training was shown to enhance patients' performance in both motor timing (movement synchronization, tapping) and in perceptual timing (duration discrimination, beat detection in music) tasks, supporting the idea that coupling gait to rhythmic auditory cues in PD relies on a neuronal network engaged in both perceptual and motor timing. In their Commentary, Mattei et al. also discuss the role of motor, cognitive, and speech networks on emotion recognition through music in PD.

*Music-supported training* (MST) using musical instruments can improve motor recovery of arm movements after stroke (Rodríguez-Fornells et al., 2012; Altenmüller and Schlaug, 2015). Van Vugt et al. extended the application of MST to the social domain by comparing stroke patients who played synchronously (together group) and who played one after the other (in-turn group). Both groups showed improvements in fine motor control and tapping and reductions in depression and fatigue, but the in-turn group showed greater improvement in fine motor skills, suggesting that stroke patients may benefit from learning through observation in MST. In two stroke patients with chronic unilateral spatial neglect, Bodak et al. assessed the impact of a music intervention that involved making sequential goal-directed actions in the neglected space by playing scales and melodies on chime bars from right to left. The patients demonstrated short- and long-term improvement on visual cancelation tasks, indicating that active music-making with a horizontally aligned instrument may help neglect patients attend more to their affected side.

## Music, learning, and memory

The interactions between music and learning and memory were explored in older adults and in patients with medial temporal lobe damage, multiple sclerosis (MS), stroke, and Alzheimer's disease (AD) in six papers. Using fNIRS, Ferreri et al. investigated whether music listening can improve episodic memory and modulate prefrontal cortex (PFC) activity during memory encoding in older adults. Compared to a silent background, upbeat music facilitated source-memory performance, and decreased dorsolateral PFC activity bilaterally, suggesting music can help older adults in memory encoding by modulating prefrontal activity in a non-demanding fashion. The long-term neural impact of music listening on stroke recovery was studied by Särkämö et al. Using voxel-based morphometry, they showed that compared to verbal stimuli (audio books) and standard rehabilitation, listening to music daily after an acute left hemisphere stroke increased gray matter volume in a network of prefrontal (superior frontal gyrus) and limbic (anterior cingulate cortex, ventral striatum) areas linked to better cognitive and emotional recovery. The results suggest that a musically enriched recovery environment can induce fine-grained neuroanatomical changes in the recovering brain.

The use of music as a mnemonic aid in MS and AD patients was investigated by two groups. In a behavioral and EEG study, Thaut et al. compared the learning of spoken and musical (sung) word lists in MS patients. Compared to the spoken condition, patients in the music condition showed overall better verbal recall and better word order memory coupled with stronger bilateral learning-related frontal synchronization, suggesting that a musical mnemonic recruits stronger oscillatory network synchronization in prefrontal areas in MS patients during word learning. Moussard et al. explored the potential of music to facilitate motor learning in healthy older adults and AD patients. The participants learned sequences of meaningless gestures accompanied either by music or metronome and done either in synchrony with the experimenter or after the experimenter. In healthy controls, musical accompaniment had no impact but synchronization helped learning. In contrast, in AD patients, musical accompaniment improved learning but synchronization interfered with retention, indicating that music may act as a mnemonic for motor sequence learning in AD.

Music-related learning in patients with medial temporal lobe damage and memory impairments was also studied by two groups. Using fMRI, Alonso et al. tested the modulatory influence of the hippocampus on neural adaptation to song melodies and lyrics. Compared to healthy controls, patients with left hippocampal sclerosis showed reduced adaptation effects to repeated lyrics and melodies in lateral temporal lobe regions, indicating that the integrated representation of lyrics and melodies is likely tied to the integrity of the left medial temporal lobe. Valtonen et al. present a case study of learning novel musical pieces by patient LSJ who was a skilled amateur violist before becoming profoundly amnesic after extensive bilateral hippocampal and medial temporal lobe damage caused by encephalitis. Three novel pieces of viola music were introduced, two of which LJS practiced playing and one which LJS did not train. Relative to the control piece, LSJ showed significant pre- to post-training improvement in the practiced pieces, which was retained in a longitudinal follow-up. These findings demonstrate that non-hippocampal structures can support complex musical learning.

## Responsiveness to music in neurological disorders

The emotional and cognitive responsiveness to music in children with severe neurological illness, adults with autism spectrum disorder (ASD), and patients with a disorder of consciousness (DOC) were explored in four papers. Using behavioral measures and EEG, Bringas et al. studied the effects of a music therapy program focusing on attention and communication training through music in children with severe neurological illness (e.g., cerebral palsy). Compared to a control group receiving standard rehabilitation, the music therapy group improved on neuropsychological status, especially on attention and communication, coupled with neuroplasticity indexed by an enhanced MMN response to phonemic changes in frontal and cingulate regions. Using fMRI, Gebauer et al. investigated the neural correlates of emotion recognition in music in high-functioning adults with ASD and healthy controls. Although both groups engaged similar neural networks during processing of emotional music, ASD individuals showed increased activity to happy vs. sad music in dorsolateral PFC and in rolandic operculum/insula, indicating increased cognitive processing and physiological arousal to emotional music in ASD.

O'Kelly et al. compared EEG, heart rate variability, respiration, and behavioral responses of healthy subjects with brain injured DOC individuals in vegetative (VS) or minimally conscious (MCS) states to different types of music (live preferred music, improvised music entrained to respiration, disliked music), white noise, and silence. Part of the VS and MCS patients were clearly responsive to preferred music as shown by both blink responses and increased EEG amplitude in frontal alpha or theta bands, indicating that music-based approaches are potentially useful as prognostic indicators and rehabilitation methods in the DOC patients. Rollnik and Altenmüller provide a comprehensive overview of the use of music therapy in neurological rehabilitation of patients with coma and DOC. They conclude that although DOC patients seem to show emotional processing of auditory information and a musically enriched environment setting may have therapeutic value, more research with clearly defined patient cohorts, standardized intervention protocols, valid clinical outcome measures, and longer and more extensive monitoring is still needed in the DOC population.

## Novel sound-based technological advances

Recent technological developments were introduced in three papers. Niu et al. studied the feasibility of a new speech synthesizer that utilizes the myocontrol of limb muscles recorded with EMG to drive the synthesis of intelligible speech. Using this device, both healthy subjects and dyskinetic CP patients were able to learn to generate English vowels, some of which were correctly identifiable by naive listeners. In the future, this approach may provide a “virtual voice” with both intellectual and social-emotional content to individuals with severe speech-motor disorders. Loui et al. presented a novel method for diagnosing seizures in epilepsy, based on the sonification of EEG data. They found that after a short training period subjects could successfully distinguish seizures from non-seizures using the auditory modality alone. Eventually, EEG sonification may help in managing, predicting, and ultimately controlling seizures using biofeedback. Another method focusing on sonification was introduced by Scholz et al. They presented a novel portable sonification device suitable for real-time 3D sonification of arm movements and explored optimal spatial mapping parameters of tone pitch and brightness. A learning experiment in healthy older persons showed that mapping pitch on the vertical axis and brightness on the horizontal axis seems an optimal constellation for motor sonification. Ultimately, movement sonification may provide an efficient and motivating way to rehabilitate gross-motor arm skills in hemiparetic stroke patients.

## Author contributions

All authors listed, have made substantial, direct and intellectual contribution to the work, and approved it for publication.

### Conflict of interest statement

The authors declare that the research was conducted in the absence of any commercial or financial relationships that could be construed as a potential conflict of interest.
